# Identifying quality responses using an analysis of response times: the RTcutoff function in R

**DOI:** 10.3389/fpsyg.2024.1479249

**Published:** 2024-11-11

**Authors:** Georgios Sideridis, Mohammed Alghamdi

**Affiliations:** ^1^Boston Children’s Hospital, Harvard Medical School, Boston, MA, United States; ^2^National and Kapodistrian University of Athens, Athens, Greece; ^3^Department of Self-Development Skills, King Saud University, Riyadh, Saudi Arabia

**Keywords:** response time, rapid guessing, psychometrics, test validity, response accuracy, CUMP method, RTcutoff function, R function

## Abstract

**Introduction:**

The present study aims to develop an R function to develop and visualize thresholds that describe the response time of individuals concerning their sample. The function utilizes the cumulative proportion correct (CUMP) approach, to estimate item-specific time threshold, which originated in the work of Guo and his colleagues. Besides the CUMP approach, the present function presents response time profiles on a measure using the mean of the sample and + 1SD times so that it can discern between thoughtful engagement and processing with an item (termed problem-solving behavior) and rapid responding, guessing, and disengagement with the test. The advantage of the CUMP model utilized here is that it simultaneously engages both response time and response correctness to establish thresholds that differentiate engaged from disengaged participants.

**Methods:**

Given data on a measure of reading comprehension for students in Saudi Arabia (*n* = 494) using the Progress in International Reading Literacy Study (PIRLS 2021) international assessment, high and low-achieving individuals that engaged in different behavior patterns were identified and plotted against their sample.

**Results and conclusion:**

Results pointed to the importance and necessity of the RTcutoff function to identify variable forms of engagement that have implications for person-score validity but also have implications for test validity and the need to increase measurement precisio.

## 1 Introduction

Finding cutoff values in the response times (RTs) is an important part of test analysis for several reasons. RTs are not only to point out just how quickly a test taker responds but also provide valuable information for exploring cognitive processes during test taking. The analysis of RTs may aid the classification of individuals as being engaged in problem-solving behaviors versus “rapid guessing” (RG, Deribo et al., 2022). Given that rapid guessing (RG) could have a significant effect on test scores and the instrument in general, it is essential to identify participants whose responses do not reflect true and valid states of their skills and competencies, who also jeopardize test score validity and reliability (DeMars, 2007). The present line of research suggests the necessity to discern engagement with a test as reflecting RG or genuine problem-solving behavior (Persic-Beck et al., 2022).

RTs, in particular, are a useful measure for identifying disengaged responses, an important component of measuring test engagement (Lee and Jia, 2014). The difference between rapid response guessing and authentic engagement as well as with a slower non-rapid response might not reflect the full extent of interaction with the content related to items (Ulrich, 2010). Very brief RTs and RG behavior consistent with disengagement from the test material have oftentimes been observed (MacPherson and Akeroyd, 2014), in both high-stakes and low-stakes testing conditions. Thus, the accurate identification and interpretation of RG behaviors is crucial to determine the level that they threaten the quality and thus validity of test scores (Bereby-Meyer et al., 2002).

In addition, the identification of RG is crucial in maintaining test security and validity, especially in computerized adaptive testing (Deribo et al., 2021). Identifying prior rapid-guessing behavior as well as establishing threshold RTs are efforts that need to be taken to anticipate ways of improving the validity and reliability of test scores (Nagy et al., 2022; Wise 2017; Wise and DeMars, 2009). Establishing response-time thresholds reflecting an arbitrary proportion of correct responses will give information about the frequency with which RG occurs and whether it can systematically bias test outcomes (Alfian et al., 2023).

RT modeling in psychometric models can be used to adjust examination difficulty levels based on examinees’ effort, especially when low-stakes test-taking situations are part of the environment with widespread unmotivated testing takers. The effort-moderated item response theory model is an approach that accounts for differing levels of effort in test performance (Wise et al., 2009), which according to Soland (2018) increases validity in measurement and identifies unmotivated students. Besides the information that RTs carry about cognitive processes, RT measures can be also useful for identifying behaviors like RG or thoughtful engagement with a task and its demands. Time and response knowledge-based models could be used to detect RG behavior (Lu et al., 2019; Yang, 2007). In addition, RG detection in response to data is necessary to protect the validity of testing procedures (Yang, 2007). Moreover, substantive results based only on conventional Item Response Theory (IRT) models might be compromised in psychoeducational and psychological assessments involving timed tests administered to the examinees with rapidly guessing behavior; by applying mixture models for responses as well as RTs, a more accurate assessment could probably be performed (Lu et al., 2019). In summary, the sophistication of mixture modeling by including RTs may elucidate distinct ability and nonability groups that serve as a useful alternative perspective on test performance (Sideridis et al., 2022). However, an understanding of the speed-ability relation and limitations clouding test outcomes should be discussed as this reveals a problem in ensuring fairness in assessments (Deng and Rios, 2022). In other words, the range of possible wild guess variability is such that it can contaminate or suppress variance and potentially mitigate value added by differential RG effects during test assessments (Deng and Rios, 2022).

In sum, the identification of cutoffs in RT is crucial across a variety of fields from psychology to medicine and education to emergency services. Thus, RTs can give clues regarding different cognitive processes and behavior patterns (like indiscriminating fast answers) or at the other end of slow and thoughtful response patterns. An analysis of strategy employed through analyzing RTs may point to individuals who behave in unexpected ways such as in the form of RG, wandering but not thoughtfully engaging with the task, or giving up overall. The magnitude of such effects may compromise a test’s psychometric qualities and specifically test validity. The proposed R function developed here utilizes one of the most prominent methods to compute critical RT thresholds (Guo et al., 2016) and is described next.

### 1.1 Introducing the CUMP method for estimating response time (RT) thresholds

Guo et al. (2016) proposed an RT threshold model for detecting rapid-guessing behavior on test items using both RT and response accuracy (RA) information (i.e., correct/incorrect responding). To set item-specific RT thresholds, the authors used a cumulative proportion correct (CUMP) approach. For each item, they plotted the CUMP of the correct response option as a function of RTs. The CUMP curve starts at the chance level (e.g., 0.25 for a 4-option item) for very short RTs and converges to the overall item difficulty as RTs increase. The authors proposed using the RT value where the CUMP curve reaches the chance level as the threshold for any given item. An important advantage of the methodology over previous models is the inclusion of item difficulty levels. The CUMP method relies on the relationship between RT and response accuracy (correctness) (Equation 1). When the relationship is weak, the precision of the threshold will most likely decrease and even approach zero when RTs exceed the user-defined threshold for guessing (usually placed at 0.25).

More specifically, the method is based on the cumulative proportion of correct responses accumulated up to time *t*. The threshold for each item is defined as the point where the CUMP curve for the correct option intersects the chance level of success. The formula for the CUMP is as follows:


(1) $$C_{j}=\max\left\{t:CUMP_{j}\left(t\right)\leq g\right\}$$

where *g* is the chance probability of success. This method extends the previous RTRA (response time and response accuracy) approaches by using cumulative data to address the issue of sparse observations at short RTs. The procedure likely provides a more objective way to determine the RT threshold for detecting RG compared to the subjective visual inspection used in the RTRA method. Guo et al. (2016) also added that the specification of upper and lower limits in RT thresholds likely accommodates varying levels of item difficulty, contributing to the robustness of the procedure over alternative models (Caplin and Martin, 2016; Sie et al., 2015; Starns, 2021; Verdonck et al., 2020). They added that the method reduces subjectivity, it is not labor-intensive, and it can account for sparse data unless the instrument under scrutiny is brief. For ease of interpretation, the proposed R function includes cutoff RT values at the mean of the sample per item, and + 1SD. Purposefully, visuals were not provided at -1SD as RTs are not normally distributed, thus, estimates at -1SD and beyond could potentially take on negative values. However, negative values in RTs are not within the natural limits of response time that must be positive.

### 1.2 Importance of the present R function

One goal of the present study was to make accessible the (CUMP) approach via an easy-to-use R function so that aberrant response patterns based on available RTs can be identified. The present R function can distinguish rapid guessers from conscientious responders. Examinees who guess rapidly show test behavior that contradicts the assumed IRT measurement model. Their scores are therefore probably invalid and can jeopardize the validity of the measurement instrument. Having the present “screening” tool is essential for keeping the scores meaningful and valid for interpretation purposes (Guo et al., 2016). Furthermore, because the R function easily differentiates between engaged and disengaged responders, this identification may be confirmed with other indicators of aberrant responding so that analytical approaches to test validity may exclude them from those tests. The present function presents a visual inspection procedure to detect rapid-guessing behavior as thresholds are computed from latency distributions along with item difficulty information (Guo et al., 2016). Thus, it is suggested that the present function can be used in different forms of assessments, in educational and psychological testing, so that RG, engagement, and disengagement are evaluated and visualized.

### 1.3 The RTcutoff function in R: applications

The RTcutoff function was designed to provide a plotting facility for the Guo et al. (2016) methodology, supplemented with additional competing thresholds such as the mean and + 1SD. The reader can “source” the R code from this GitHub address: https://github.com/GS1968/RTcutoff, in R, and then read RTs and responses as two separate comma-delimited files (see Documentation file in GitHub). The data for the present illustration came from the Progress in International Reading Literacy Study (PIRLS 2021) and the participating sample from the Saudi Arabia Kingdom. The function was applied to a reading comprehension measure with 9 items, selected so that the measure would be unidimensional and internally consistent (Cronbach’s alpha = 0.71; Omega = 0.73; Marginal Reliability = 0.73). Data were dichotomous and the function can be used with dichotomous items as the plot facility includes success at the item level which is possible using a correct-incorrect scoring system. Furthermore, the function utilizes as inputs comma-delimited data files or comma-separated files (i.e., .csv). Missing data on responses or response times will leave those estimates in the plot empty, thus, missing data are accommodated. Table 1 displays the item parameters from fitting a 2-parameter logistic model (2PL) to the data. Items were re-ordered from easy to difficult so that they could be easily applied to the R function. As shown in the table, all items had discrimination parameters close to or larger than 1 showing adequate discriminant ability. Furthermore, item difficulties ranged between −0.930 and + 1.620. Omnibus model fit was good with the M_2_ statistic (alternative to the chi-square test) being non-significant [M_2_(27) = 31.510, *p* = 0.250] and the Root Mean Squared Error of Approximation (RMSEA) being less than the recommended cutoff value of 0.05 (RMSEA = 0.02). Thus, the 2PL model fits the data well to the reading comprehension scale. The full-scale items are shown in Appendix B. Example comprehension questions were “Who is Sam?” or “Where does Sam put his book when he finishes?.” Data can be freely downloaded from the IEA here: https://pirls2021.org/data/, however, the exact dataset used in the present study is included along with the function and a documentation file in this GitHub repository.^1^ The function was applied to *n* = 494 Saudi students and, for illustration purposes, four selected participants are discussed in detail next to demonstrate the value of the function to identify differential engagement patterns.

**Table 1:** Item parameters of the reading comprehension scale.

Items	*a*	*s.e.*	*b*	*s.e.*
RE41M09	1.120	0.170	−0.930	0.150
RE41M05	1.900	0.280	−0.850	0.110
RE41M07	1.490	0.210	−0.530	0.100
RE41M10	2.230	0.330	−0.370	0.080
RE41M13	2.420	0.370	−0.110	0.070
RE41M15	0.350	0.110	0.000	0.270
RE41M02	1.030	0.160	0.080	0.110
RE41M11	0.760	0.140	0.710	0.170
RE41M14	1.810	0.370	1.620	0.190

After Fitting the 2PL Item Response Theory Model. a = item discrimination; b = item difficulty; s.e. = standard error of measurement.

**Figure 1:**
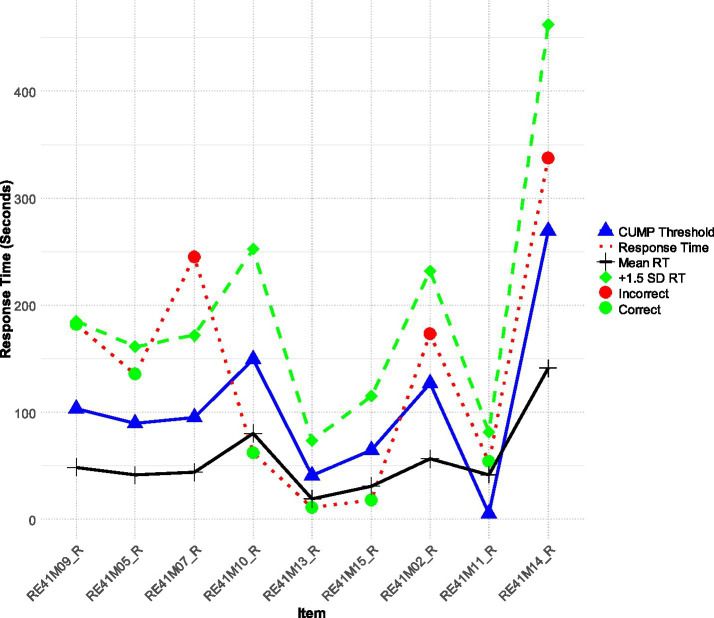
Participant 216 is an above-average achiever who takes their time on easier tasks and then spends a lot more time on more difficult items (theta = 0.341).

Figure 1 presents participant 216 as they appear in that row of the database. Participant 216 showed an above-average ability in reading comprehension (theta score = +0.341 logit, i.e., about a 0.35 standard deviation above the mean of the sample the participant belonged to) and presented effortful performance with the most difficult items in terms of their failure. That is, items 3, 7, and 9 resulted in failed attempts (see red circles versus green circles defining item successes or failures) and, interestingly, this person allocated maximum effort in these items. Thus, we would classify this person as a persistent and effortful test-taker, as task difficulty did not result in decrements in their effort. The determination and persistence shown to challenging questions, in particular, is one reason why including RT is critical when interpreting test-taker behavior and engagement (Guo et al., 2016).

**Figure 2:**
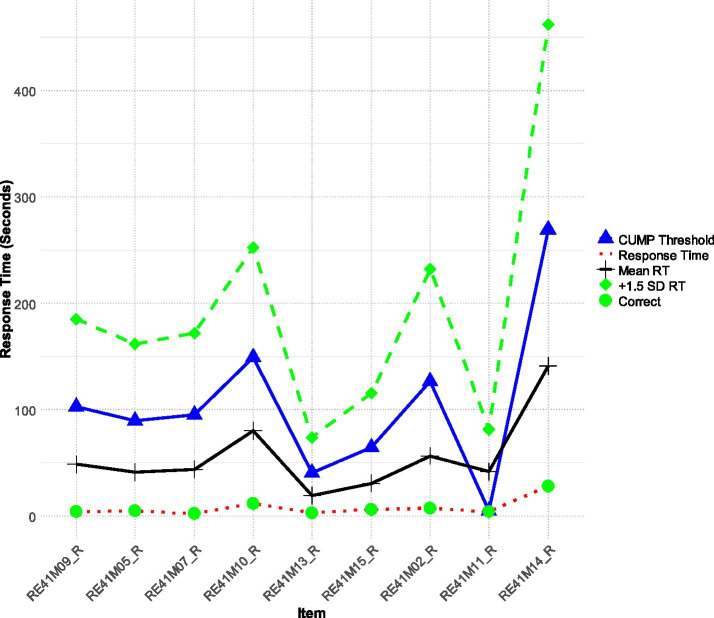
Participant 21 is a rapid responder but a very high achiever with a theta score equal to 1.71.

Participant 21 (see Figure 2) identified as a very high achiever (+1.71 logits of ability - theta) presented quick response rates to tests without compromising accuracy. This performance is interesting as this participant does not follow the typical pattern of quick responding on easy tasks and slower responding on difficult tasks; instead, participant 21 demonstrated fast RTs, which were consistently below both the CUMP and + 1SD thresholds, regardless of item difficulty. Thus, participant 21 showcased a very competent reader with the capability to process information quickly and accurately. The behavior of Participant 21 contrasts with the conventional wisdom that RG practices diminish performance. Their analysis of RTs also highlights the need to interpret RTs more cautiously, especially for high-ability individuals (Guo et al., 2016) so that they would not be flagged as false positive cases of RG.

**Figure 3:**
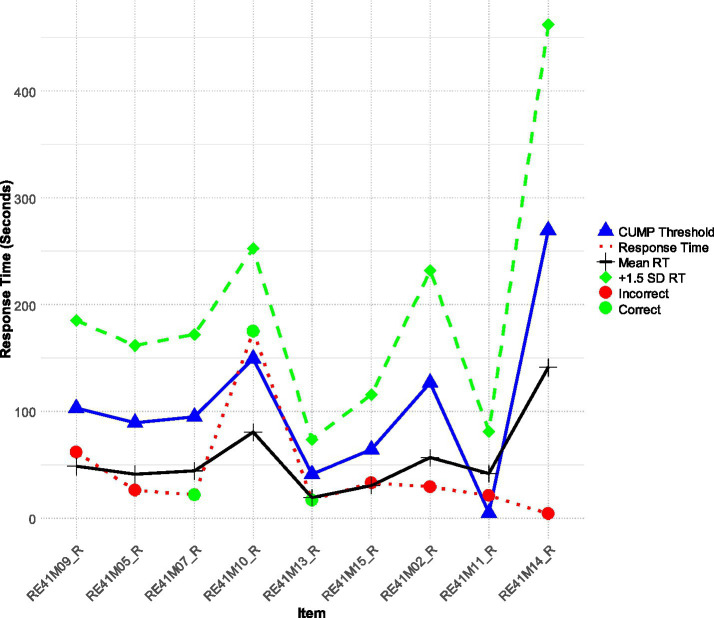
Participant 27 is a low achiever who has likely given up after initial successful attempts and after facing a first failure.

Participant 27, (low achiever −0.221 logits), demonstrated an interesting pattern of engagement (Figure 3) with quick times on the first 3 easy items in which this person was successful. Then participant 27 faced item 4 which was a little more challenging and failed. Following that failure, engagement times dropped dramatically and so was achievement with items 5 through 9 being incorrect. We can only speculate that Participant 27 may have become frustrated or fatigued and resorted to guessing quickly on the later items. This behavior is indicative of a loss of motivation and could potentially inform interventions. In other words, being able to identify such patterns is key as it can inform understanding of the challenges faced by low-achieving students and ultimately help in designing supports that will keep them engaged across a full test cycle (Nagy et al., 2022).

**Figure 4:**
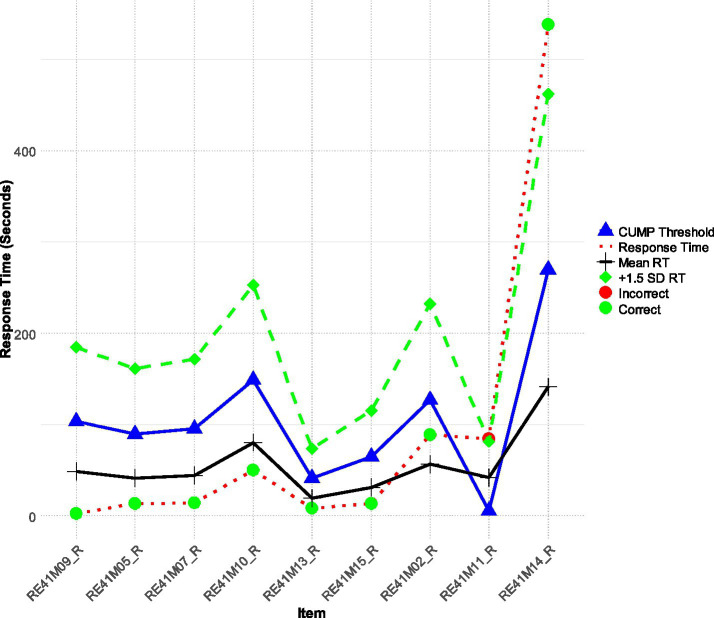
Participant 169 is characterized as being an engaged and persistent high achiever.

Last, Figure 4 describes a person (Participant 169) who was a high achieving student with a theta score of +1.405 logits, exemplified an ideal test-taking strategy. This participant allocated time efficiently across items, spending less than the average time on easier items (1 through 6) and dedicating significantly more time than the mean RT and the CUMP threshold for the more difficult items (items 8 and 9). The RTs for the difficult items were above both the CUMP and + 1SD thresholds, indicating a high level of persistence and cognitive effort. We consider the present participant to reflect an optimal engagement pattern that reflects strategic thinking as the participant allocates required cognitive resources depending on test-item difficulty levels. Such behavior not only enhances the accuracy of test results but also stresses the participant’s robust problem-solving skills and endurance. Understanding this ideal engagement pattern can inform strategies to support other test-takers in managing their time and effort effectively.

**Figure 5:**
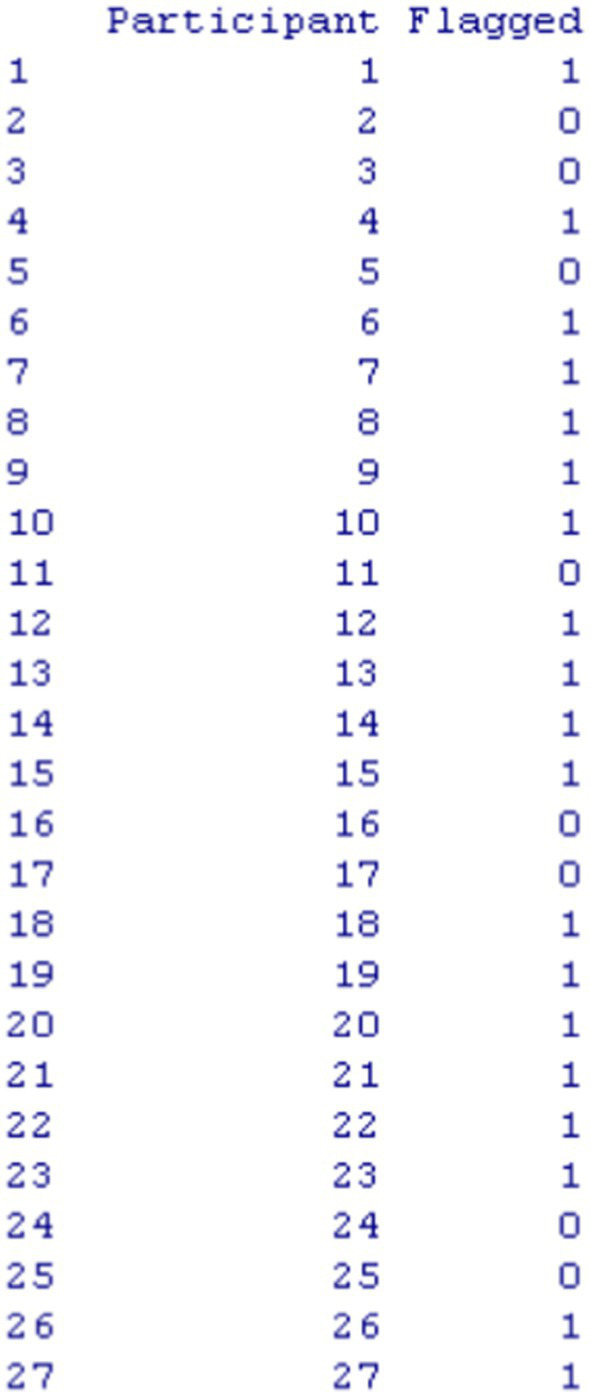
The output of the RTCutoff function that shows flagged responders.

Furthermore, to make the function efficient and useful when samples are large, we implemented a flagging procedure so that individuals with aberrant engagement times could be identified. Figure 5 displays an output using the first 26 participants with the flagging column representing aberrant response time participants with values of “1” versus “0.” Flagging involved the following criteria: (a) a person’s response time on an item deviates from the mean CUMP threshold for that item by ±1.3 standard deviation, and (b) the pattern of response time exceeding ±1.3 standard deviation occurs in 50% or more of the items of the measure. The selection of a 1.3 standard deviation in either tail of the distribution represents the lowest or highest (slow or fast responding) as being representative in about 10% of the response time distribution. Thus, the selection of ±1.3 standard deviation aligns with aberrance being reflected in 5% of the sample using a one-tailed test. We understand that this is an arbitrary cutoff but is based on distributional criteria and a low percentage that usually signals that a person is different from the respective sample or population they belong to. As shown in Figure 5, participants 21 (Figure 2) and Participant 27 (Figure 3) selected earlier were identified as being aberrant responders using the flagging criteria.

### 1.4 Conclusions, limitations, and final remarks

The present R function estimates RTs and is aimed at setting good cutoff criteria to know about behavior which infers motivation through engagement. This function uses three different cutoffs and plots people on those threshold values to determine how a single person is engaging in relation to the rest of the participants in the sample.

Though promising, however, the current R function and the choice of the CUMP methodology have their drawbacks. One challenge with the CUMP method involves addressing a sparse data problem at short RTs which can reduce reliability in determining thresholds. Even though the cumulative approach lessens this issue to a certain extent, it is still one of its limitations that needs to be considered (Guo et al., 2016). Second, the current approach assumes fixed chances of success for rapid guesses by the number of response options. This simplification may not illustrate the full complexity of guessing behavior in all testing scenarios for which a fixed-chance success rate may not be appropriate (Bulut et al., 2023). This assumption could be improved by further refinement (Guo et al., 2016). Third, the method might not be as effective for items that are either too easy or very hard and therefore did not have their cumulative proportion correct cross the chance level within a reasonable number of trials. Fourth, the CUMP method assumes that students who rapidly guess should have a cumulative proportion correct near the chance rate. When this assumption is violated (e.g., if correct responses are higher than chance even for short times), the threshold may indeed approach zero. This limitation of the CUMP method, particularly with very easy or very difficult items, has also been discussed by Soland (2018). Fifth, we acknowledge that the current function primarily focuses on RG, and the issue of wandering behavior deserves more attention. Bulut et al. (2023) emphasize that long RTs associated with wandering may also result in incorrect answers, potentially skewing the CUMP-based threshold, thus, in the presence of wandering, the function may not accurately estimate a proper threshold value. In such instances beyond RG evaluation, the interested reader may consider alternative methodologies (e.g., Guo et al., 2016).

Future studies could investigate item and test-taker characteristics that suggest a more adaptive threshold detection strategy. This could be done with machine-learning approaches where thresholds are adjusted differently per context. By leveraging machine learning algorithms, researchers can tailor threshold adjustments based on specific contexts, enhancing the adaptability and accuracy of threshold detection mechanisms (Zhang, 2024) by adjusting sampling uncertainties (Huber, 2024). Machine learning algorithms have demonstrated the capability to analyze diverse factors, such as physiological data, performance metrics, and external conditions, to identify patterns and correlations, which can inform the optimization of threshold detection strategies (Barbour et al., 2018; Shamstabar, 2024; Şahin and Colvin, 2020). It is also easy to see how the current function could be extended, for example, from educational assessments to other domains such as psychological testing, medical diagnostics, and employee assessment. In each, there can be variations in how to adjust the methodologic approach for distinct response behaviors per domain accounting for unique contextual and personal factors.

## Data Availability

The original contributions presented in the study are included in the article/Supplementary material, further inquiries can be directed to the corresponding author.

## Footnotes

^1^https://github.com/GS1968/RTcutoff

## Appendix A

R code to estimate cutoff values in RTs after sourcing the *RTcut1.R* function.

Both the person response data and the response time data need to be sorted so that the same participant is on the same row in both files.

#Example

flag_data <− calculate_flags_and_plot(“path to .csv response time data,” “path to .csv data,” participant_row = 5)

## Appendix B

Reading comprehension questions and their coding in PIRLS 2021, comprising the reading comprehension instrument. These 9 selected items include the scoring of a single correct response although additional items (not included here) are scored using partial credit schemes:

RE41M02: The Library belongs to Sam.

RE41M05: Where does Sam put his book when he finishes it?

RE41M07: What do the children think of Sam’s book?

RE41M09: Why is Sam invited to the meeting?

RE41M10: How does Sam feel when he reads the note?

RE41M11: Which words from the note does Sam not understand?

RE41M13: What does Sam put inside the box?

RE41M14: The girl is surprised when she looks in the box.

RE41M15: Why does Sam put a pile of pencils next to the box?
