# Supplementary material for: Identifying quality responses using an analysis of response times: the RTcutoff function in R
Source: Front Psychol. 2024 Nov 11;15:1479249. doi: 10.3389/fpsyg.2024.1479249 (PMC11587929; doi:10.3389/fpsyg.2024.1479249)

**How to Apply the RTcut1 Function from Github**

Please follow the following steps to apply the function using the user-defined datasets. First visit the following github address: https://github.com/GS1968/RTcutoff

1. When visiting the github address you will see the following:
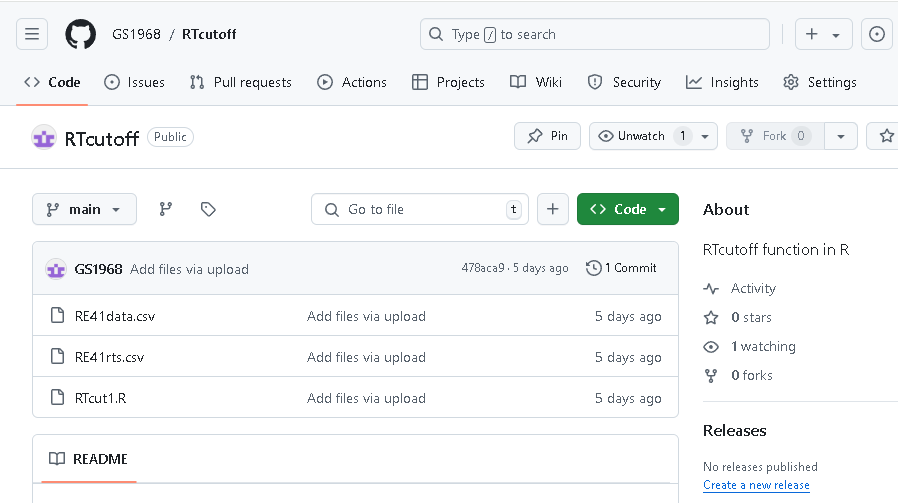

2. Download the documentation file (Documentation RTcutoff. Function.docx) to see each one of the steps
3. Download the R function and the datasets from the above address by clicking on each one of them and then select download as shown below. Please save to the directory of interest to you and mark that directory so that you can locate this information from R.
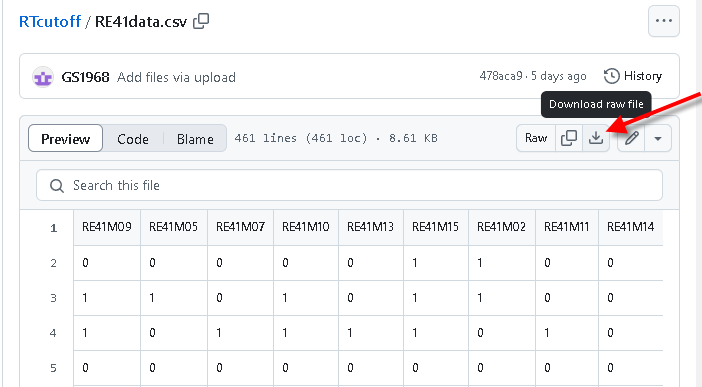

4. The datafiles for your data will need to look as follows for the response vectors and the response times in .csv format:

Data (RE41data.csv)


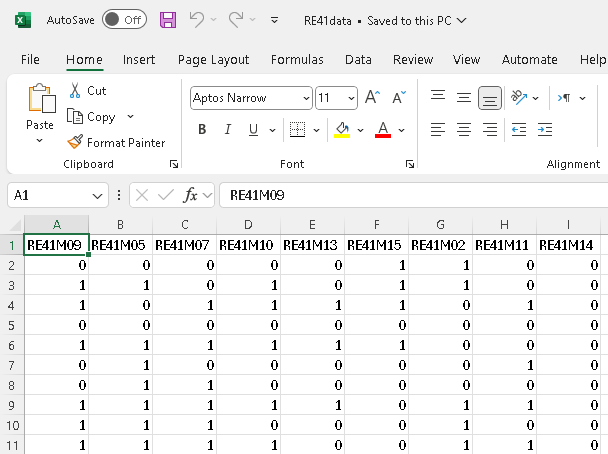


Response times (RE41rts.csv)


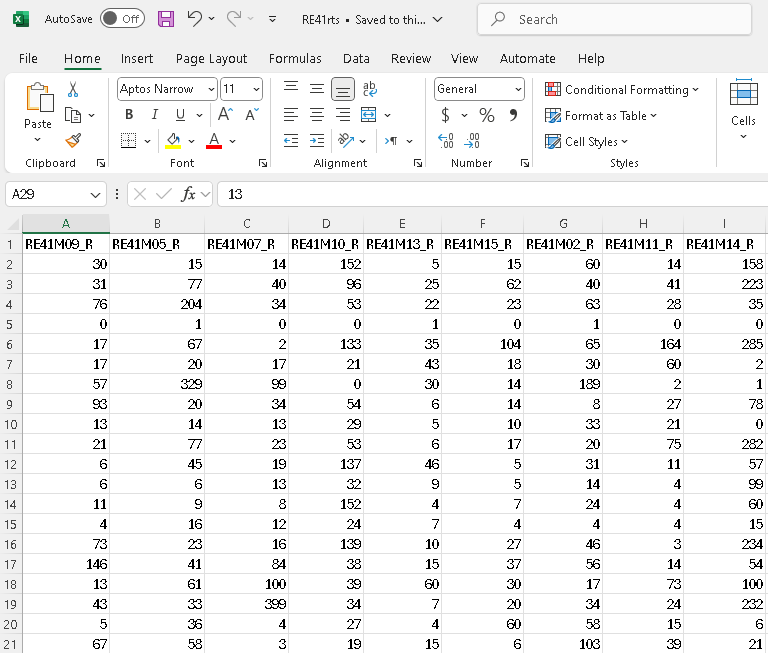


1. Source the R function in R: Open a recent R version (the function was applied in R.4.4.1) and source the R code as shown below:
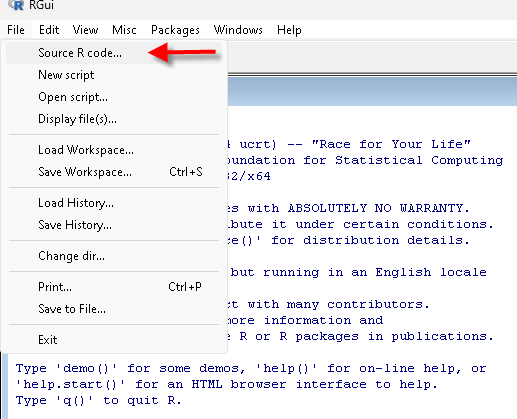


Select the RTcut1.R function from the directory you earlier saved all files and source the code. If R does not source the code check the directory and also re-start and run R as an administrator. If it runs properly, you will be asked to select a mirror to download prerequisite packages as shown below:
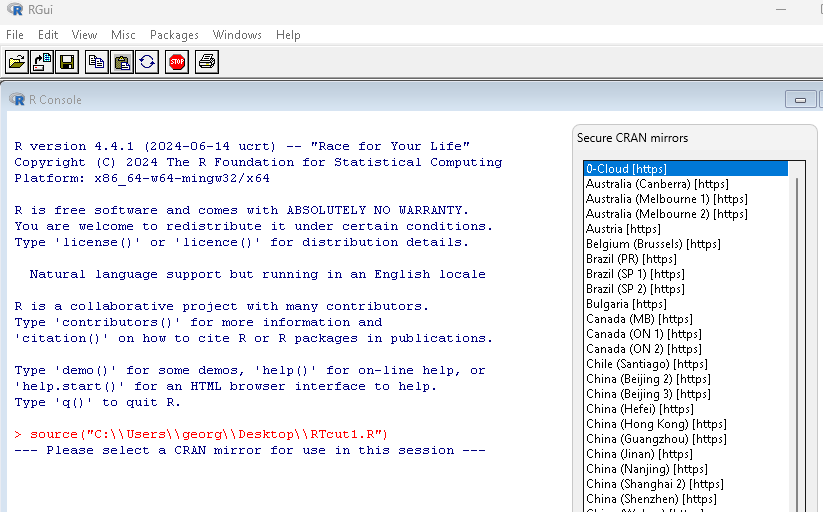


1. After sourcing the function, then you need to call the function and have it read the two datasets with the response times and response vectors. These data must have been earlier downloaded from you and be in a specified directory that you will need to include its path when calling the function. To call the function use the following command to run the function with participant 1 (in row 1) but you can repeat with any single participant you wish to view their response time performance:

calculate_thresholds_from_csv("C:\\Users\\Georg\\Desktop\\RE41rts.csv", "C:\\Users\\Georg\\Desktop\\RE41data.csv", participant_row = 1)

1. After applying all the above you will see the results for participant 1 as follows:


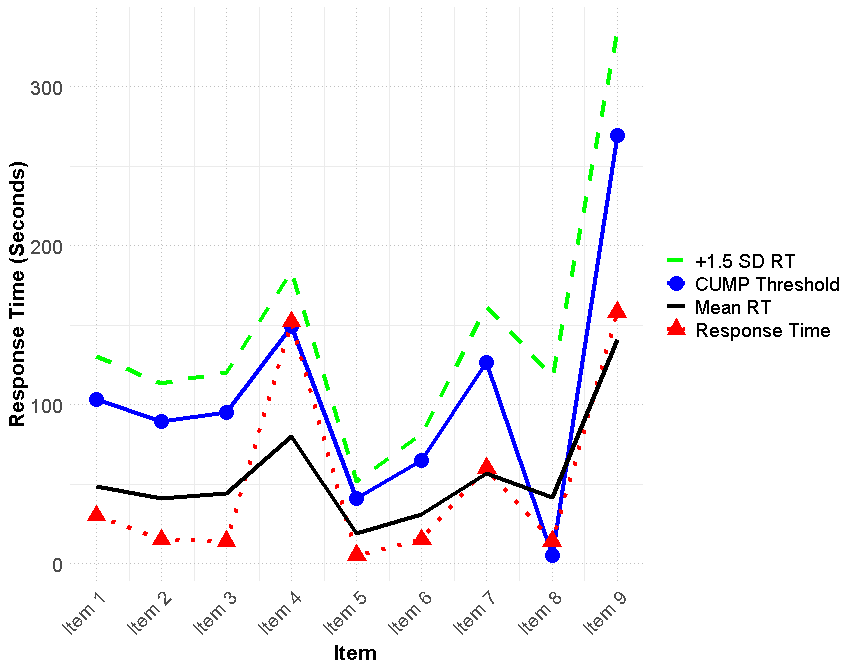

Supplement: Supplementary file 1 [file Table_1.DOCX]
